# Supplementary figures and images for: Metabolic reconstitution of germ-free mice by a gnotobiotic microbiota varies over the circadian cycle
Source: PLoS Biol. 2022 Sep 20;20(9):e3001743. doi: 10.1371/journal.pbio.3001743 (PMC9488797; doi:10.1371/journal.pbio.3001743)

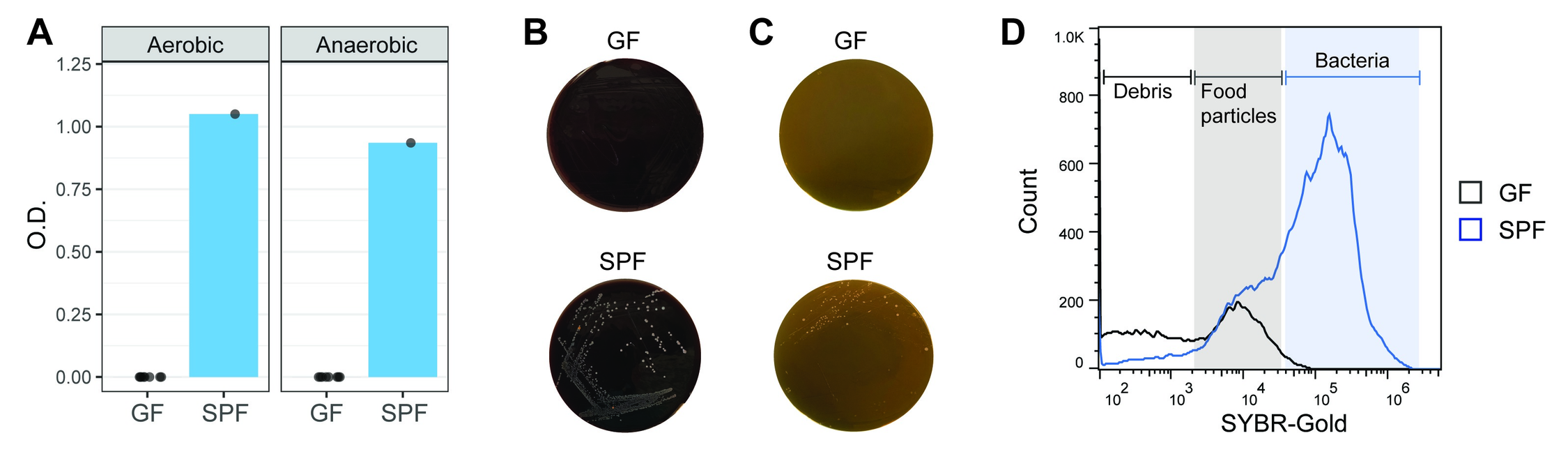

Supplement: S1 Fig — (A) OD measurement of BHIS liquid cultures incubated overnight in aerobic and anaerobic conditions. (B-C) Representative (B) BHI-blood and (C) YPD plates streaked with GF and SPF cecum content and incubated for 3 d. (D) Representative histograms bacteria flow cytometry plots of PBS, GF, and SPF cecum content stained with SYBR Gold. Data underlying this figure are supplied in S1 Data. GF, germ-free; OD, optical density; SPF, specific-opportunistic-pathogen-free. (TIF) [file pbio.3001743.s001.tif]

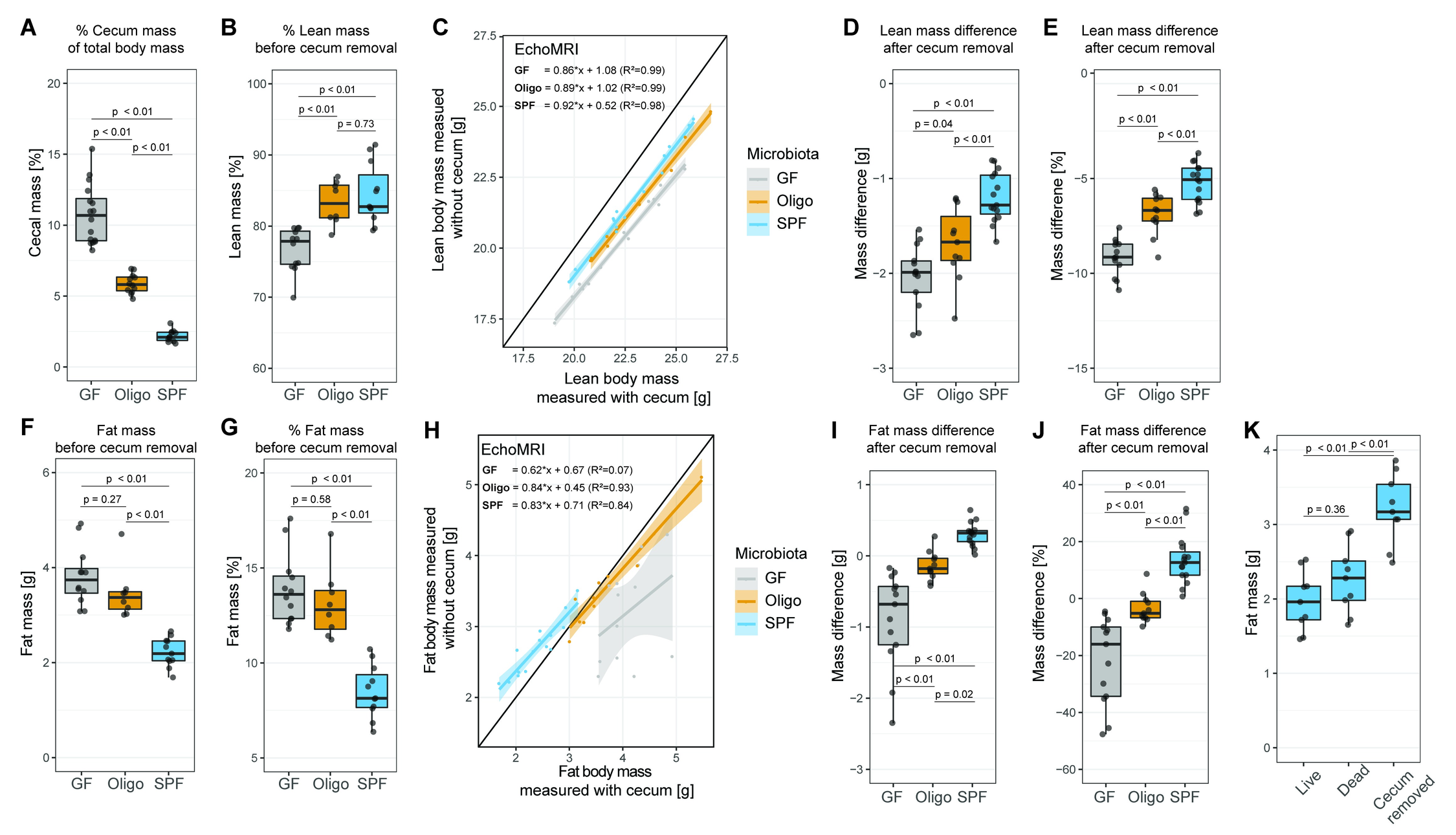

Supplement: S2 Fig — (A) Cecal mass (tissue including luminal content) as percentage of total body mass (N of mice per group: GF = 16, OligoMM12 = 12, SPF = 11) (B) Percentage of lean body mass before cecum removal. (C) Lean body mass estimated by EchoMRI with and without cecum. Measurements were taken on live animals (x-axis) and dead animals after cecum dissection (y-axis). Equations show simple linear regression for estimating lean mass without cecum based on lean mass with cecum; in brackets adjusted R-squared. (D) Lean mass difference after cecum removal. (E) Lean mass difference after cecum removal as percentage of lean mass before cecum removal. (F) Fat body mass acquired by EchoMRI before cecum removal. (G) Percentage of fat body mass before cecum removal. (H) Fat body mass estimated by EchoMRI with and without cecum. Measurements were taken on live animals (x-axis) and dead animals after cecum dissection (y-axis). Equations show simple linear regression for estimating fat mass without cecum based on fat mass with cecum; in brackets adjusted R-squared. (I) Fat mass difference after cecum removal. (J) Fat mass difference after cecum removal as percentage of lean mass before cecum removal. (K) Fat mass measured by EchoMRI in live, dead, and cecum-removed SPF mice (n = 9). Number of mice per group in all figures unless otherwise specified: GF = 13, OligoMM12 = 11, SPF = 15. p-values obtained by Tukey’s honest significance test. Data underlying this figure are supplied in S1 Data. GF, germ-free; SPF, specific-opportunistic-pathogen-free. (TIF) [file pbio.3001743.s002.tif]

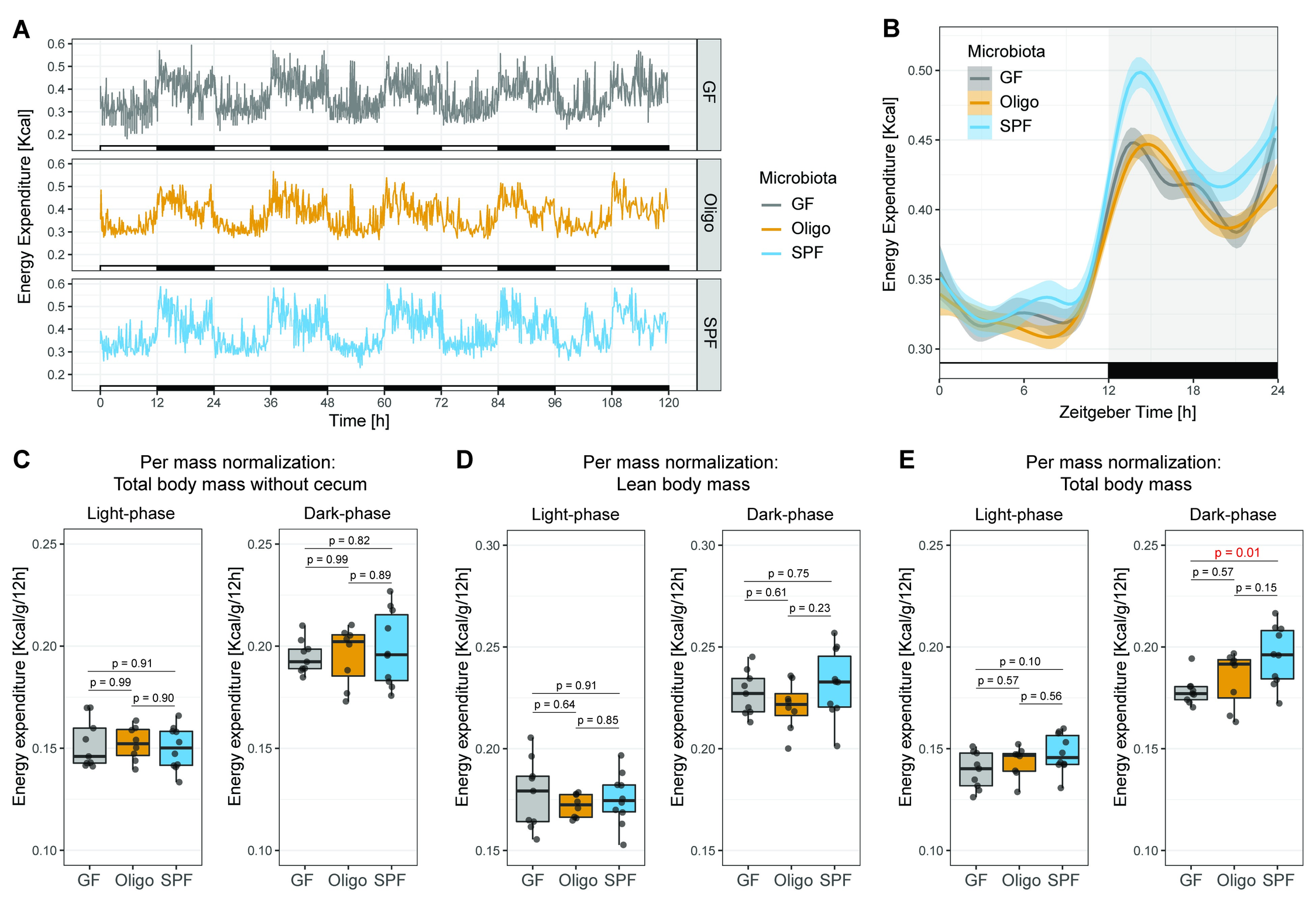

Supplement: S3 Fig — (A-B) Comparison of circadian changes in energy expenditure (without normalization) among GF, OligoMM12, and SPF C57B6/J mice. (A) Circadian variation in average energy expenditure per time point and (B) overlayed curves obtained by smoothing function of data obtained every 24 min per mouse over 10 d. (C-E) Energy expenditure values obtained by “classical” ratio-based normalization methods (dividing energy expenditure values per phase by mass). (C) Area-under-curve after normalization by total mass after cecal dissection. (D) Area-under-curve after normalization by lean body mass (EchoMRI). (E) Area-under-curve after normalization by total body mass before cecal dissection. Number of mice per group in all figures unless otherwise specified: GF = 9, OligoMM12 = 8, SPF = 10. p-values obtained by Tukey’s honest significance test. Data underlying this figure are supplied in S1 Data. GF, germ-free; SPF, specific-opportunistic-pathogen-free. (TIF) [file pbio.3001743.s003.tif]

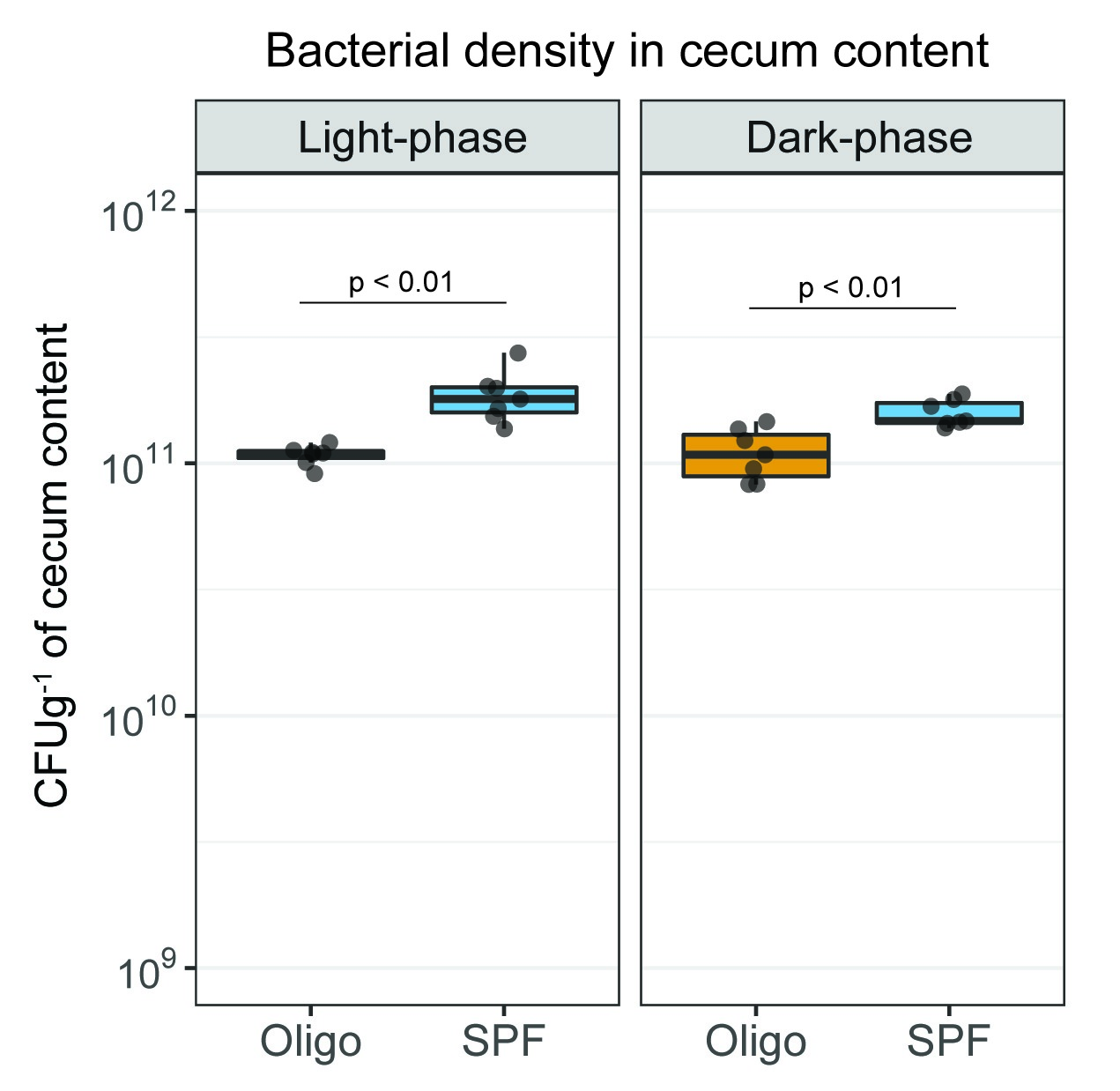

Supplement: S4 Fig — (A) Bacterial density in cecum content of OligoMM12 and SPF mice during the light and dark phase quantified by flow cytometry. Data underlying this figure are supplied in S1 Data. (TIF) [file pbio.3001743.s004.tif]

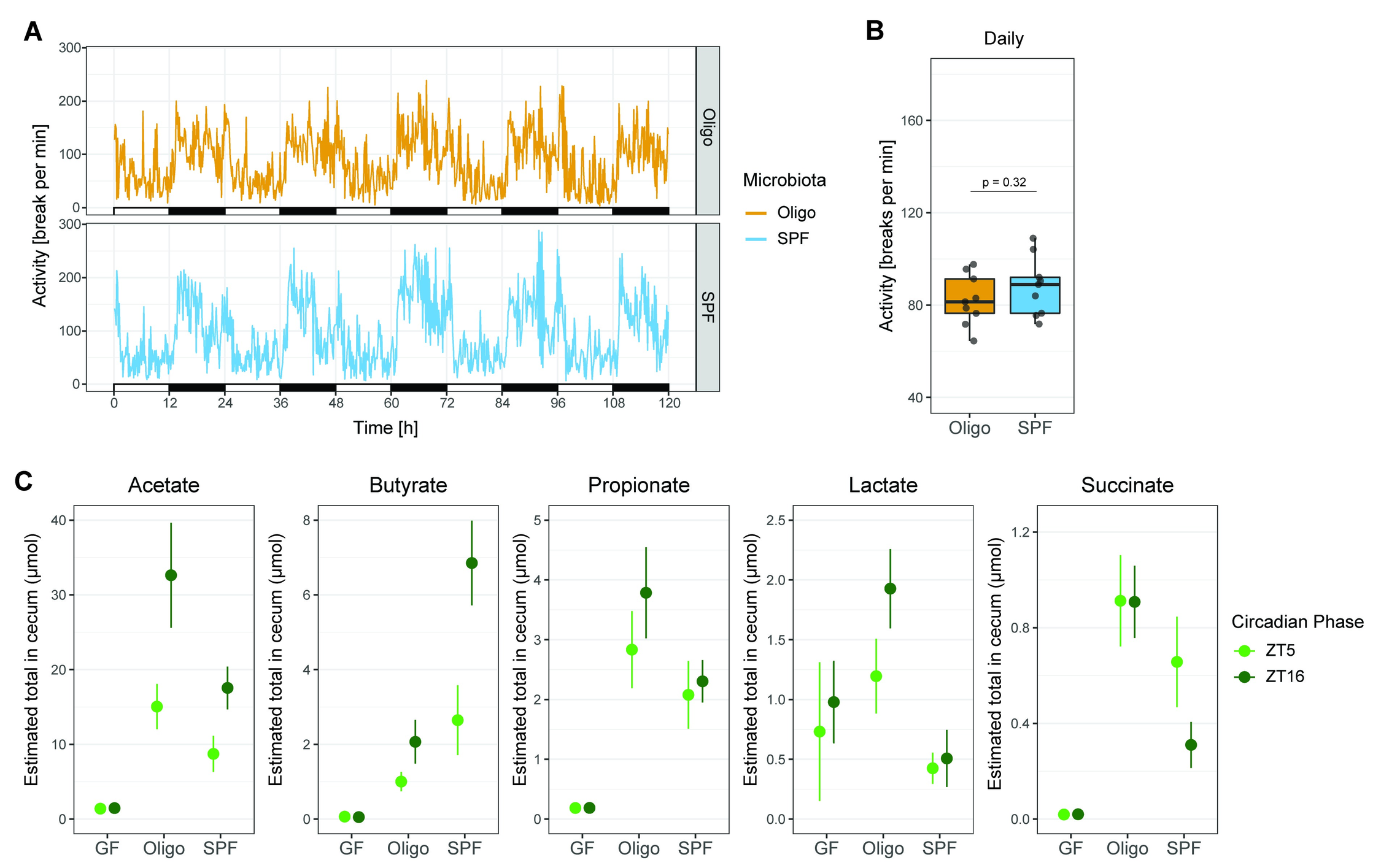

Supplement: S5 Fig — (A-B) Locomotor activity in OligoMM12 and SPF mice (n = 9 per group): (A) Circadian variation in average breaks/minute per time point. (B) Average daily breaks/minute. (C) Estimation total amount of SCFAs and intermediate metabolites by multiplying measured concentration values by the cecal mass of the group. Number represented estimate mean value ± combined standard uncertainty from measurements used for calculations. Number of mice per group in all figures unless otherwise specified: GF = 13, OligoMM12 = 12, SPF = 10. p-values obtained by Tukey’s honest significance test. Data underlying this figure are supplied in S1 Data. GF, germ-free; SCFA, short-chain fatty acid; SPF, specific-opportunistic-pathogen-free. (TIF) [file pbio.3001743.s005.tif]

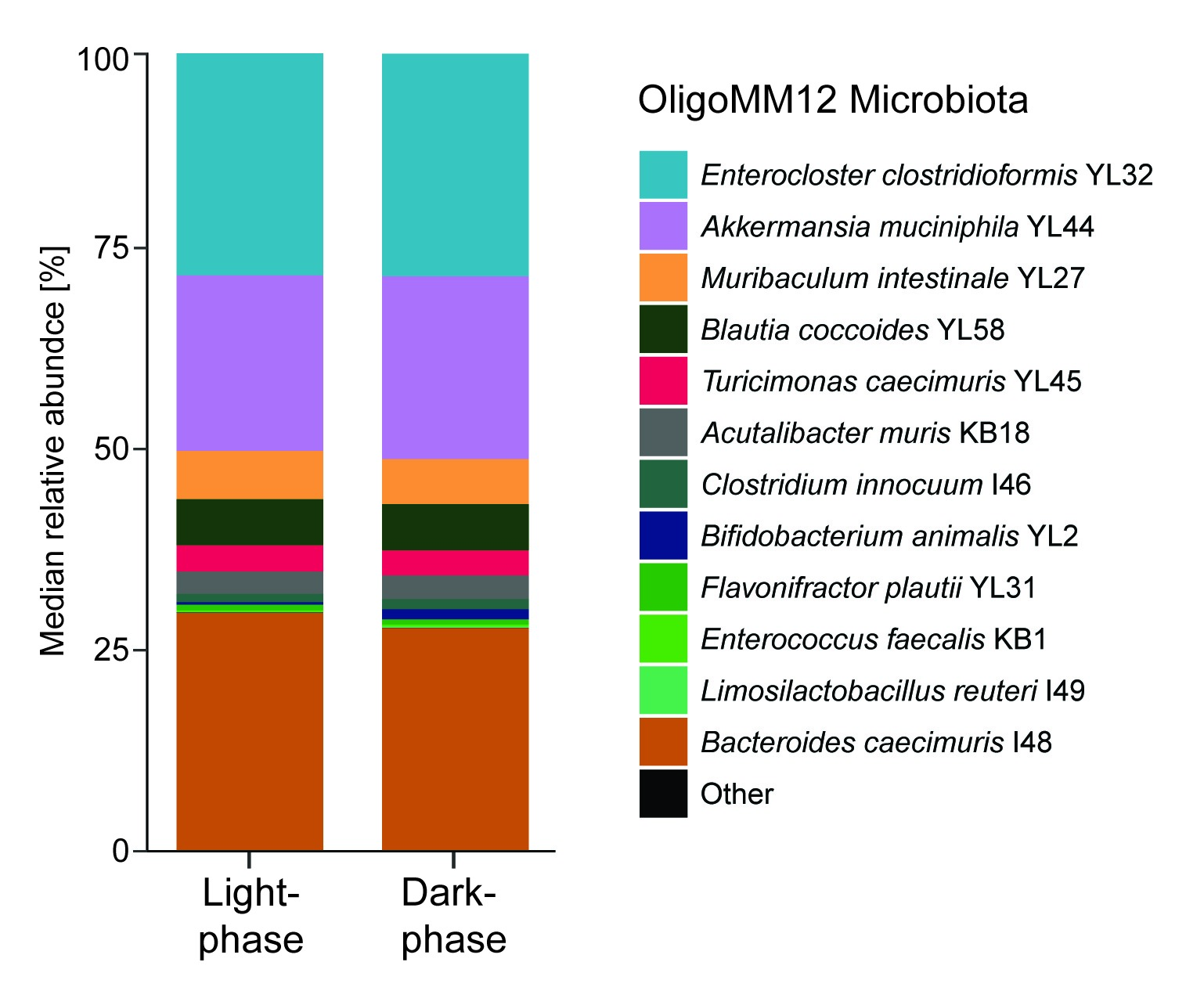

Supplement: S6 Fig — Data underlying this figure are supplied in S1 Data, and raw sequencing data are publicly available on the European Nucleotide Archive (ENA) under the Project ID PRJEB53981. (TIF) [file pbio.3001743.s006.tif]

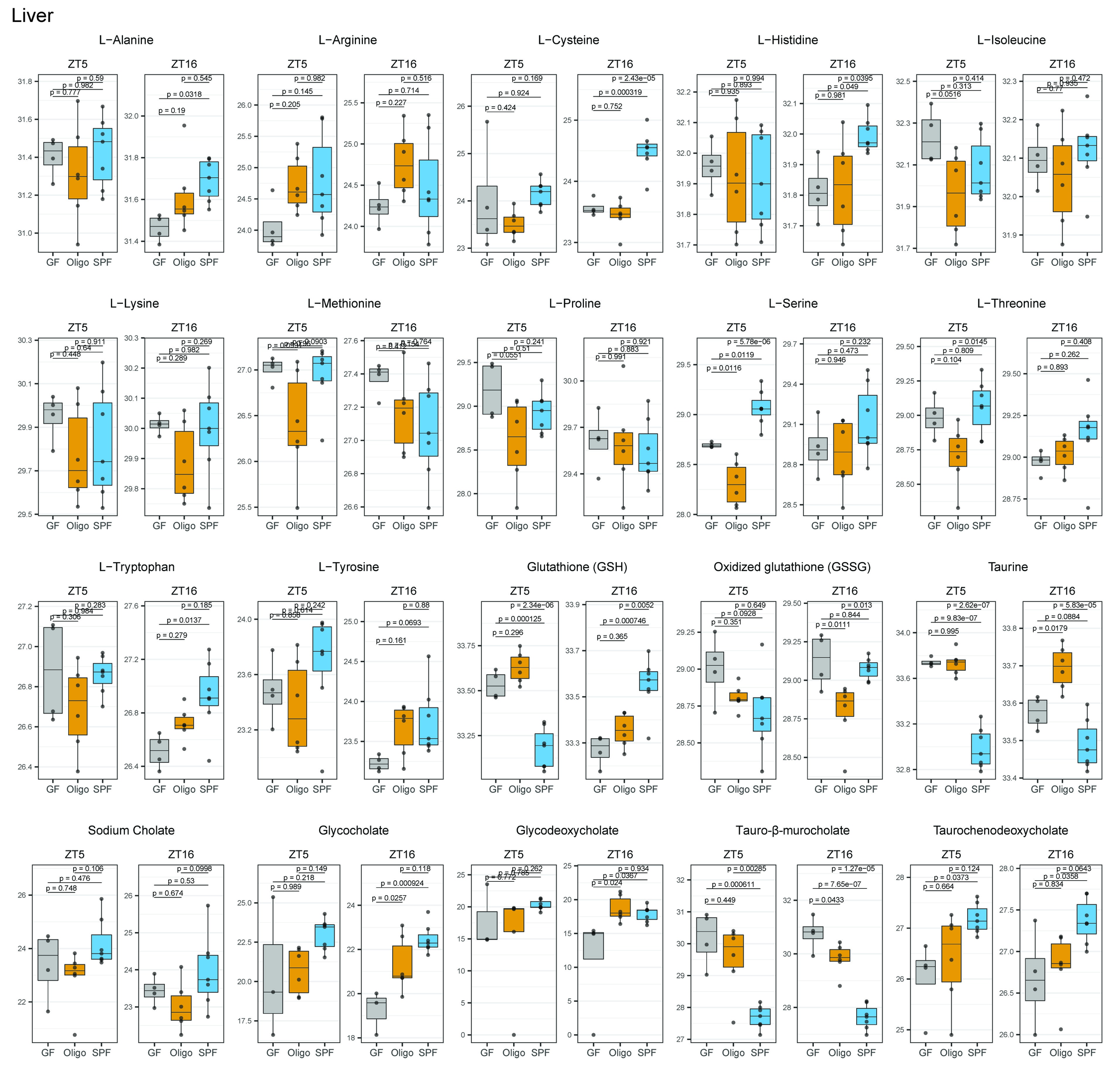

Supplement: S7 Fig — Manually curated list of compounds obtained by targeted peak extraction from differentially expressed pathways in liver samples during the light phase (ZT 5) and dark phase (ZT 16). p-values obtained by Tukey’s honest significance test after log2 transformation of area value. Number of mice per group: ZT5: GF = 4, OligoMM12 = 6, SPF = 7; ZT16: GF = 4, OligoMM12 = 6, SPF = 7. Data underlying this figure are supplied in S1 Data. GF, germ-free; SPF, specific-opportunistic-pathogen-free; UPLC/MS, ultraperformance liquid chromatography coupled with mass spectrometry; ZT, Zeitgeber time. (TIF) [file pbio.3001743.s007.tif]

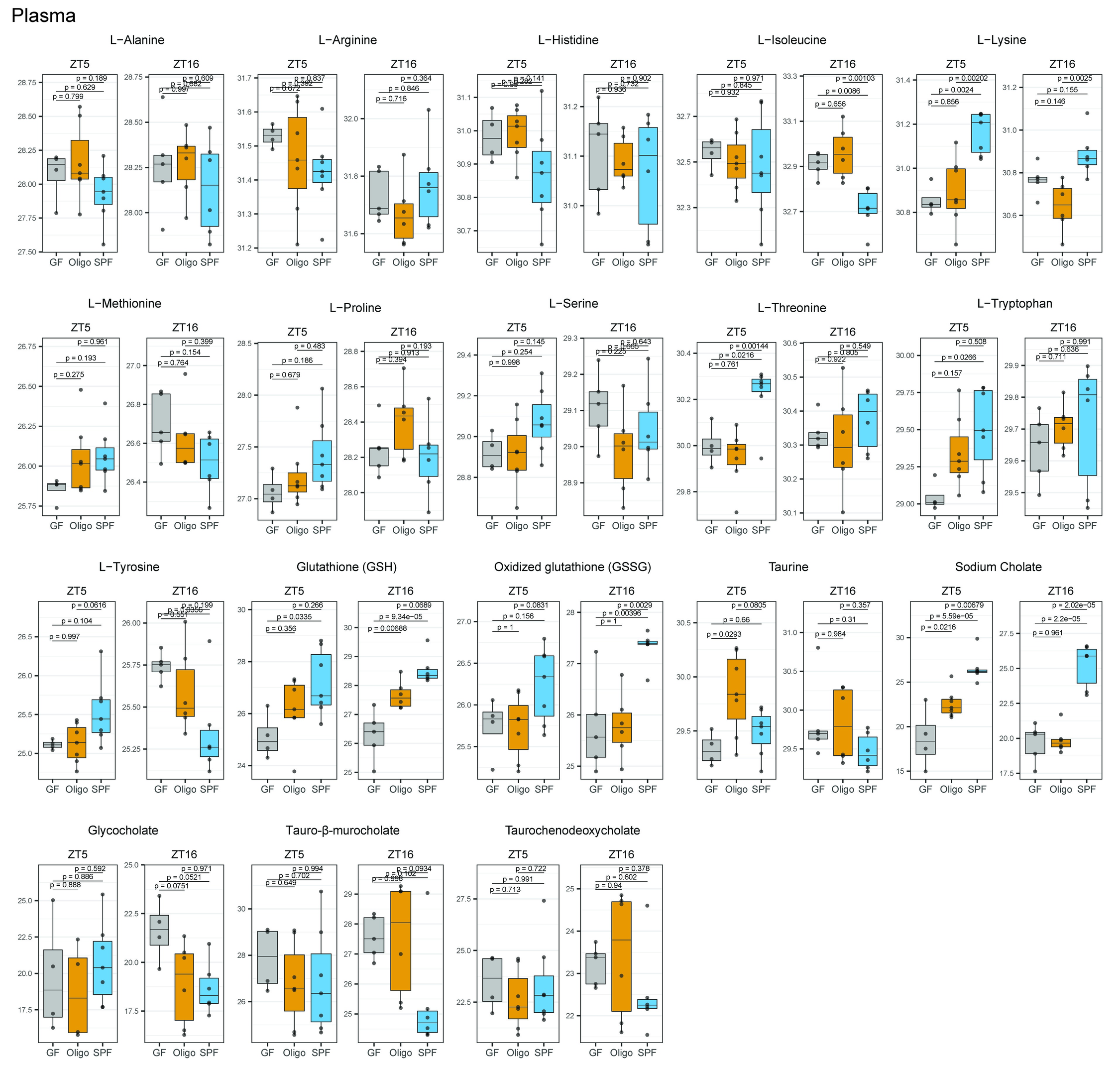

Supplement: S8 Fig — Manually curated list of compounds obtained by targeted peak extraction from differentially expressed pathways in plasma samples during the light phase (ZT 5) and dark phase (ZT 16). p-values obtained by Tukey’s honest significance test after log2 transformation of area value. Number of mice per group: ZT5: GF = 4, OligoMM12 = 7, SPF = 7; ZT16: GF = 5, OligoMM12 = 6, SPF = 6. Data underlying this figure are supplied in S1 Data. GF, germ-free; SPF, specific-opportunistic-pathogen-free; UPLC/MS, ultraperformance liquid chromatography coupled with mass spectrometry; ZT, Zeitgeber time. (TIF) [file pbio.3001743.s008.tif]
