# Supplementary material for: Metabolic reconstitution of germ-free mice by a gnotobiotic microbiota varies over the circadian cycle
Source: PLoS Biol. 2022 Sep 20;20(9):e3001743. doi: 10.1371/journal.pbio.3001743 (PMC9488797; doi:10.1371/journal.pbio.3001743)
Supplement: S1 Table — Table indicates compound name, KEGG entry number, type of column was used for UPLC and if the peak ID matched the retention time and MS2 spectra identified with the chemical standard in liver and plasma samples. Data of all compounds in liver and plasma samples during the light phase (ZT 5) and dark phase (ZT 16) available in S1 Data. UPLC/MS, ultraperformance liquid chromatography coupled with mass spectrometry; ZT, Zeitgeber time. (DOCX) [file pbio.3001743.s009.docx]

**List of metabolites identified by targeted peak extraction in the UPLC/MS data**.

| **Compound** | **KEGG Entry** | **Column** | **Matched peak ID in Liver** | **Matched peak ID in Plasma** |
| --- | --- | --- | --- | --- |
| (R)-3-hydroxybutanic acid | C01089 | RP Negative | Yes | No |
| 5-Oxoproline | C01879 | AMIDE Positive | Yes | Yes |
| Arachidonic acid | C00219 | RP Negative | Yes | Yes |
| Betaine | C00719 | AMIDE Positive | No | Yes |
| beta-Murocholic acid | C17726 | RP Negative | Yes | Yes |
| Cholic acid | C00695 | RP Negative | Yes | Yes |
| Citrulline | C00327 | AMIDE Positive | Yes | Yes |
| Cortisol | C00735 | RP Positive | Yes | No |
| Creatine | C00300 | AMIDE Positive | Yes | Yes |
| Creatinine | C00791 | AMIDE Positive | No | Yes |
| Cystathionine | C02291 | AMIDE Positive | Yes | Yes |
| Deoxycholic acid | C04483 | RP Negative | No | Yes |
| Docosapentaenoic acid | C16513 | RP Negative | Yes | Yes |
| Glutathione (GSH) | C00051 | AMIDE Positive | No | Yes |
| Glycine | C00037 | AMIDE Positive | Yes | Yes |
| Glycocholic acid | C01921 | RP Positive | Yes | No |
| Hexadecanedioic acid |  | RP Negative | Yes | Yes |
| Hippuric acid | C01586 | RP Negative | Yes | Yes |
| L-(+)-Ornithine | C00077 | AMIDE Positive | Yes | Yes |
| L-4-Hydroxyproline | C01157 | AMIDE Positive | Yes | Yes |
| L-Alanine | C00041 | AMIDE Positive | Yes | Yes |
| L-Arginine | C00062 | AMIDE Positive | Yes | Yes |
| Lauroylcarnitine |  | RP Positive | Yes | Yes |
| Leucine | C00123 | RP Positive | Yes | Yes |
| L-Glutamic acid | C00025 | AMIDE Positive | Yes | Yes |
| L-Histidine | C00135 | AMIDE Positive | Yes | Yes |
| Linoleic acid | C01595 | RP Negative | Yes | Yes |
| L-Isoleucine | C00407 | RP Positive | Yes | Yes |
| L-Lysine | C00047 | AMIDE Positive | Yes | Yes |
| L-Methionine | C00073 | AMIDE Positive | Yes | Yes |
| L-Proline | C00148 | AMIDE Positive | Yes | Yes |
| L-Serine | C00065 | AMIDE Positive | Yes | Yes |
| L-Threonine | C00188 | AMIDE Positive | Yes | Yes |
| L-Tryptophan | C00078 | RP Negative | Yes | Yes |
| L-Tyrosine | C00082 | RP Negative | Yes | Yes |
| Myristic acid | C06424 | RP Negative | Yes | Yes |
| N,N-dimethylglycine | C01026 | AMIDE Positive | Yes | Yes |
| N-Acetylmethionine | C02712 | RP Positive/Negative | Yes | Yes |
| Oxidized glutathione (GSSG) | C00127 | AMIDE Negative | Yes | Yes |
| Pantothenic acid | C00864 | RP Positive | Yes | Yes |
| Pipecolinic acid | C00408 | RP Positive | Yes | Yes |
| Serotonin | C00780 | RP Positive | Yes | Yes |
| Succinic acid | C00042 | RP Negative | Yes | Yes |
| Taurine | C00245 | AMIDE Negative | Yes | Yes |
| Taurine-beta-murocholic acid |  | AMIDE Negative | Yes | Yes |
| Taurochenodeoxycholic acid | C05465 | AMIDE Negative | Yes | Yes |
| Tetradecanedioic acid |  | RP Negative | Yes | Yes |
