# Supplementary material for: Metabolic reconstitution of germ-free mice by a gnotobiotic microbiota varies over the circadian cycle
Source: PLoS Biol. 2022 Sep 20;20(9):e3001743. doi: 10.1371/journal.pbio.3001743 (PMC9488797; doi:10.1371/journal.pbio.3001743)
Supplement: Attachment — Submitted filename: Response_to_reviewers_13Jun2022.docx [file pbio.3001743.s011.docx]

Response to reviewers.

We would like to thank all three reviewers for their insightful and useful comments on the manuscript – all three reviews were of a very high quality, and this is hugely appreciated by all authors. We have taken all suggestions into account and now include additional data on sterility (Suppl. Fig. 1), % body composition (Suppl. Fig. 2), microbiota density (Suppl. Fig. 4), Locomotion (Fig.3 and Suppl. Fig. 5) and microbiota composition (Suppl. Fig. 6). Additionally further analysis of our metabolomics data is now included in Suppl. Fig. 7 and 8.

To facilitate the re-review process, all changes to the text are highlighted in yellow in a “track changes” version and the page/paragraph number is listed in the point-by-point response below.

Reviewer #1: Hoces et al. present a careful examination of how OligoMM12 mice differ from SPF and GF mice in terms of bodyweight (Fig. 1), energy balance (Fig. 2), RER and SCFAs (Fig. 3), and metabolomics of liver and plasma (Fig. 4). They use a clever setup to monitor calorimetric measurements under sterile conditions, which allows them to address some of the long-standing questions in the field of metabolic host-microbiota interactions.
Among the most interesting outcomes are the finding that OligoMM12 mice have larger fat depots than both SPF and GF mice (Fig. 1G), that GF mice successfully compensate for their lack of microbiota-derived calories by eating more (Fig. 2), and that GF, SPF, and OligoMM12 mice have approximately equivalent energy expenditure (Fig. 2B) and absolute energy extraction (Fig. 2K), but SPF mice extract a higher percentage of calories from food (Fig. 2L). This manuscript contributes to an improved understanding of the ways in which OligoMM12 mice do and don't mimic SPF mice.

- Thank you for these supportive comments. All authors feel that this review was of very excellent quality and has greatly helped us to improve the manuscript. We thank you for your time and effort.

Please find below several questions and suggestions that I hope will help improve this manuscript.

* Line 124: The authors present absolute lean mass in Fig. 1F, but it would be nice to see percent lean mass. For example, SPF mice seem to have the lowest body weight (Fig. 1D), but the highest lean body mass (Fig. 1F), suggesting that they have significantly higher percent lean mass. This would be an interesting finding to report. The same is true for percent fat mass, but I understand the authors' reservations about doing this given their observation that the measurement of fat mass is sensitive to cecal weight (Fig. S1E).

- In the interests of full transparency, we added a new figure showing the lean mass percentage (using lean mass before dissection Suppl. Fig 2B). Note that the lower % lean mass in germ-free mice is attributable to massive cecal water retention and not to an increased fat mass. The % fat mass read-outs, as you already suggested, are very susceptible to artefacts of cecum-content calling in the EchoMRI readouts (see tabulated values below for EchoMRI of cecum alone). These challenges are reported in the results section (Results, page 8/9) and discussed in the second paragraph of the discussion (page 18). Our overall conclusion remains that great caution is needed with the use of EchoMRI for body composition comparisons between mice with major anatomical differences, such as those seen in germ-free mice.

| Microbiota | %Fat | %Lean | % Water |
| --- | --- | --- | --- |
| GF [Mean (SD)] | 34 (4) | 22 (10) | 44 (24) |
| SPF [Mean (SD)] | 14 (8) | 41 (15) | 38 (28) |

* Line 128: It is an interesting point that EchoMRI measurements are different pre- and post-cecum dissection. In the Methods, I saw that "for a set of mice," EchoMRI was performed on euthanized mice pre- and post-cecum removal. Are all the datapoints in Fig. S1E on dead mice, or is the y-axis dead mice and the x-axis live mice? I bring this up because the inconsistency in fat mass may also be related to comparing live v. dead mice. At least one publication reports EchoMRI inconsistencies when comparing live to dead animals (PMID: 21152249).

- This is an interesting point and clearly an additional complication in EchoMRI analysis. To accurately address this, we carried out a set of analyses on a group of mice that were measured while still alive, after euthanasia, and after cecum removal. The new data is presented in Suppl. Fig. 2K and described in the results (page 9). Clearly there is a small difference in the values for live versus dead mice (consistent with the previous studies (suggested reference and PMID 14742854)), but this is a considerably smaller effect than the difference associated with cecum removal in SPF mice. This seems to be an artefact of the EchoMRI readout, and we cannot mechanistically explain this. We could not find reports of EchoMRI measurements after major anatomical changes such as cecum removal. Therefore, we recommend caution in the use of EchoMRI for fat mass measurements in mice with marked anatomical differences (i.e., enlarged cecum due to axenic status). This is discussed on p22, first paragraph.

* Line 129: In Fig. S1, I suggest changing the word "variation" to "difference." Variation made me think standard deviation and variance, but the authors are simply referring to the difference between pre- and post-cecum measurements.

- Thank you for this suggestion. We have changed the labels in what is now Suppl Fig.2D, E, I and J accordingly.

* Line 131: Fig. S1F is indeed baffling: how could there be higher fat mass when excluding the cecum? I appreciate that the authors included this puzzling result. They should discuss if this is incongruent with previously reported results.

- We obviously share your confusion here, and beyond the fact that it must be a technical artefact of the measurement system, we also cannot well explain the observation, nor can we find useful references relating to such phenomena when using EchoMRI as a readout. As suggested we have added a brief text urging caution in interpretation of EchoMRI read-outs (page 20, paragraph 1) and suggesting the use of dissected fat mass as a more easily-interpretable read-out of body fat particularly in animal groups with large differences in intestinal water retention.

* Line 134: The authors argue that directly weighing dissected fat mass is a more reliable way of estimating fat mass, given the EchoMRI weirdness they showed in Fig. S1E. However, dissection of small tissues weighing 50 to 500 mg (Fig. 1G) also seems imperfect. Very slight differences in the boundaries of tissue removal could have large effects on tissue weight. It was reassuring to read that dissection was performed while "blinded to the hygiene status of the mice," but, in my experience, the very large differences in cecum size prevent true blinding. One way to strengthen the authors' argument is to quantitatively compare their tissue weights to previously reported results (e.g., Bäckhed 2014, Suárez-Zamorano 2015), although such comparisons across studies and vivaria might be difficult.

- Thank you for highlighting this challenge. We have added a comment in the Methods section (p28, “Body composition measurements”) describing how we tried to minimize the bias on the fat depot dissection. Briefly, another investigator removed first the cecum (D.H.) before a second investigator (W.S.), blind to the hygiene level and cecum mass, performed the fat tissue dissection. This was designed as such both because of the types of analyses needing to be done on the respective tissues, and such that W.S. would be as much as possible blinded to the experimental groups.
- Thanks for the suggestion to compare our findings on dissected fat mass of GF and SPF to previous findings. This has been added to the discussion section (page 18, paragraph 2) . Both of the suggested studies showed a significant increase in the fat mass for SPF mice when compared to GF. However, the absolute masses differ considerably between the studies, likely related to the age of the animals and other factors. While we accept that we could be under-powered to detect a real difference in white adipose tissue deposits between germ-free and SPF mice, there are already a large number of animals analyzed and all due care was taken to ensure that our analysis was accurate and unbiased. We therefore stand by the conclusion that in our colony, any difference is very small. Discrepancies between this and the suggested previous reports could be attributed to a wide range of factors including the exact composition of the SPF microbiota, the composition of the mouse chow, housing conditions, genetic background, etc. and unfortunately cannot be dissected post-hoc.

* Line 150: Did the authors also try normalizing by lean body mass after subtracting the weight of the cecum? The cecum should be mostly (entirely?) lean mass, rather than fat mass, so this normalization approach would mimic the "classical normalization" of dividing by lean body mass but after adjusting for the confound of cecal weight.

- Thank you for this suggestion. In line with the suggestions of reviewer 2, we have moved most of these different normalizations to a comprehensive supplementary figure and now also include your suggestion to normalize using the total body mass after cecum removal (Suppl. Fig 3C). This gives a similar result to the per-mass normalization based on lean body mass (using the EchoMRI measurements pre-cecum removal, Suppl. Fig. 3D). The reason why these two per-mass normalizations are similar is because the cecum mass, when assessed by the EchoMRI, makes only a small contribution to the total lean mass in both GF and SPF mice (but has a huge effect on predicted fat mass). To clarify this, we provide the EchoMRI readings for cecum below:

| Microbiota | %Fat | %Lean | % Water |
| --- | --- | --- | --- |
| GF [Mean (SD)] | 34 (4) | 22 (10) | 44 (24) |
| SPF [Mean (SD)] | 14 (8) | 41 (15) | 38 (28) |

As observed in the table, approximately half of cecum content is considered as water in both GF and SPF mice. In the remaining half, a higher percentage of the cecum content is considered as fat in the GF mice. Considering the mean value of cecum mass of each group (GF = 3.0g and SPF=0.58g), in GF mice the cecum will contribute approx. 1g of what the EchoMRI considers to be “fat” compared to 0.08g in SPF, which is a huge contribution. However, the cecum will contribute 0.66g of lean mass to in GF vs 0.24g in SPF, which makes a neglectable contribution to the approx. 20 to 22g of lean mass than GF and SPF mice have. This is briefly clarified in the text (page 8, paragraph 2)

* Line 165: It is interesting and surprising that feces from GF mice have fewer calories than feces from SPF mice. I like the authors' explanation for this observation, but they should discuss it in the context of previous papers that have reported bomb calorimetry done on feces from gnotobiotic mice.

- We considered that the observed difference of approx. 0.1 Kcal/g observed between colonized and GF mice can be explained by the bacterial mass present in the feces of OligoMM12 and SPF mice. We now discuss these findings in the context of previous studies that showed similar results to ours when compared GF and SPF animals (Discussion p23, paragraph 2,, doi:10.1038/nature05414, <http://www.ncbi.nlm.nih.gov/pubmed/6834773>. Although this was previously observed to be strongly dependent on diet: doi:10.1073/pnas.0605374104.). In general, there is a good agreement between published data on germ-free versus fully colonized mice, and our equivalent data.

* Line 203: Showing that changes in food intake don't explain differences in RER is an important control. The other behavior that could affect RER is locomotion, which unfortunately doesn't seem to have been measured in this manuscript. Physical activity is known to increase RER, so it would have been nice to investigate whether differences in physical activity could explain the differences in RER. Do the authors happen to have measurements of locomotion?

- Previous data have shown, using a sterile inserted intraabdominal transmitter, that GF mice have a higher locomotor activity compared to SPF mice (Bäckhed, 2007). Unfortunately, our isolator-housed phenomaster system does not include the possibility to track activity, and the only activity-tracking system available to us is based on open cages. We reasoned that GF would get immediately contaminated in the non-filtered room-air based TSE Phenomaster system. Nevertheless, we know that the OligoMM12 colonization is stable for short time-periods and therefore these animals can be held for a few days without major contaminations. As we also observed RER differences between OligoMM12 and SPF mice, we compared locomotor activity in OligoMM12 and SPF mice using the open-cage based TSE PhenoMaster system. This data is now added to Fig 3C and Suppl Fig 5A and B. Briefly, no measurable differences were observed in locomotion between OligoMM12 and SPF mice in either the light- or dark-phase. Therefore, locomotor activity cannot explain the observed differences in RER. This is described in the results section (page 12, paragraph 3; page 13, paragraph 1) and discussed on page 22, paragraph 1.

* Line 263: In Fig. 4C-D, the most consistent hit seemed to be butanoate metabolism. Are any of the molecules in Fig. S4 related to butanoate metabolism? It could be nice to have a sentence or two (and a graph or two) zooming in on this hit.

- Thank you for the excellent suggestion. We have added 3 graphs showing compounds that we acquired by targeted peak extraction and are present in the butanoate pathway (R-3-hydroxybutanoate, succinate and L-glutamate) to Fig. 4C-F. Our pathway enrichments take into consideration differences in both intermediates and final products of the pathways but allow positive and negative comparisons to contribute to the final score. This is based on the concept that flux through these pathways may not be uniform. Nevertheless, we do observe consistent trends, for example succinate and glutamate tend to be high in OligoMM12 mice. These data are briefly described in the text (page 16/17).

* Line 355: The RER data were used to emphasize that OligoMM12 mice resemble GF mice during the day and SPF mice at night, but the metabolomic data consistently showed that OligoMM12 and GF mice were always close together (both during the day and night). Therefore, I'm not sure that "In line with the RER data" is the appropriate transition here.

- We completely agree this comment. We have modified the text and the discussion of this relationship to rather highlight the strong similarity between germ-free and OligoMM12 mice compared to SPF mice (page 15).

Praiseworthy highlights:

* Line 187: This is a nice result showing that SPF mice have microbes (not present in OligoMM12 mice) that improve energy extraction from food. This is consistent with OligoMM12 mice having a bigger cecum than SPF mice.
* Line 222: OligoMM12 mice producing much more hydrogen than the two other groups is a striking result.
* It is clear that the authors thought carefully about appropriate data normalization.

- Thank you for these comments – this is a very nice section to see in a review!

Very minor concerns:

* Line 246: Please define UPLC-MS the first time it's introduced.
* Typo in line 256: "OilgoMM12"
* Typo in line 454: "After samples all samples"

- Thank you for the editing. Typos have been corrected.

----------------------------------

Reviewer #2: Hoces, et al. present a manuscript demonstrating the effects of GF and a reduced community, OligoMM12 on energy expenditure, RER, SCFA metabolism, and metabolomics changes. In particular the true contribution of the manuscript is the ability to use metabolic cages in an isolator system, thus allowing better metabolic characterization of GF and Oligo mice. This is truly remarkable and exciting as is seeing their data confirming and challenging hypotheses of the metabolic effects on GF and reduced community systems. Moreover, the authors recognize that cyclical changes in feeding and their dynamic effects on the luminal environment and host dynamics influences their metabolic measures, which again strengthens the paper. A few reservations exist though. Better circadian characterization of the microbiome of the Oligo and SPF would have helped inform bacterial-metabolic relationships the authors are using. There was a particular focus on cecal weight (taking up two
figures as well as supplemental figures) which is not interesting since it did not affect their ultimate conclusions and distracted from other more important metabolic findings. Finally, the metabolomic results, particularly of bile acids, a far more important finding that is relevant to many groups, is essentially ignored. More detailed comments below.

MAJOR COMMENTS

1.      Though it is clear that the microbiome has cyclical dynamics in SPF mice (based on previous papers the authors have cited) and the GF mice have no microbiome dynamics (since they don't have a microbiome), what is the dynamic changes in the microbiome of Oligo mice? Could the different results observed in day vs. night be the result of shifts in relative abundance of bacteria in their reduced community? Or do they occur despite a lack of cyclical oscillations?

- Thank you for highlighting an obvious analysis that should be added! In response to this, we have carried out amplicon analysis of the OligoMM12 mice during the light and dark cycle. As you can see in the microbiota composition analysis shown in Supp. Fig. 6, with total abundance of bacteria shown in Supp. Fig. 4, there are no major shifts in species abundance (either total or fractional) between the day and night cycle in the OligoMM12 mice. We therefore conclude that the metabolic alterations are the result of changes in metabolic activity of individual strains rather than shifts in bacterial abundance. This is now described on page 14, paragraph 2.

2.      The authors present so many different versions of their EE data that the message is getting lost in their attempts to show their rigor. Though normalization to body compartments (not total body weight) is common and recommended (PMID 20103710), there needs to be more clarity as to whether their results would be different if they had used lean mass vs lean mass without cecum.  My understanding of the experimental conditions presented here is that using lean mass (as opposed to other body compartments or total body weight) makes most sense since GF and low abundance communities (e.g. OligoMM12) lead to poor nutrition states. The fact that cecal removal affects lean mass percentage and could potentially affect EE is interesting. But the authors show that their conclusions were not affected by cecum removal (Fig 2CDE). Hence, I don't really understand why this is such a big part of the paper. It seems like most of what is Fig 1 and 2 can go into supplemental figures (i.e., here are the EE results, cecal removal did not affect our conclusions) and they should focus on the main message here (that EE is not different between GF/OligoMM12/SPF).

- Thank you very much for this comment. We have selected the regression-based normalization (using lean mass and fat depots as variables) as the “most correct” readout for comparing EE between GF/OligoMM12/SPF. This figure, in addition to the quantification of energy intake and output by bomb calorimetry, focus on the main message that the EE is similar among these mice. We have moved all the other per-mass normalization variations (now also including cecum removal) to the Suppl. Fig. 3.

3.      The authors should include mouse activity data over time.

- Unfortunately, activity monitoring is not available in our isolator-housed TSE Phenomaster system. The only available activity monitoring system is based on open cages and is therefore impossible for long-term monitoring of germ-free mice that would be immediately contaminated. In order to at least partially address this, we have carried out activity monitoring of the OligoMM12 mice, which are relatively stable to major contaminations, and compared this to SPF mice. This data is now presented in Fig. 3C, as well as Suppl Fig 5A and B, and demonstrates no difference in locomotor activity between the mouse groups. This data is described on page 12/13.

4.      Given the important of bacterial bile acid modifications on host physiology, what is the effect of OligoMM12 on bile acids? The authors hint that these are different and include it in a supplementary table, but this will require far more fleshing out. Since the organisms in the OligMM12 are known, their potential effect on bile acid pool should also be known. Are bile acid changes in line with what is expected? Or are certain bile acid changes occurring at certain times? The current manuscript as written has a missed opportunity here.

- We absolutely agree that bile acid modifications in the OligoMM12 microbiota and the circadian rhythm of these modifications is an exciting topic. In fact, this is a major research focus of some of our colleagues not actively involved in this manuscript (for example, Prof. Siegfried Hapfelmeier, University of Bern, Prof. Josef Ecker, Technical University Munich, and Prof. Thomas Clavel, RWTH Aachen) and therefore our investigation of potential bile acid metabolism-related mechanisms has been deliberately kept minimal. The bile acids that could be non-ambiguously identified in our UPLC-MS analysis are now shown in Fig. 4 and Suppl. Fig. 7 and 8. While the picture that emerges is quite complex with regard to colonization and circadian rhythm, our observations are in line with published reports on the effect of microbiota presence/absence on bile acid composition. For example, both, us (Fig. 4C-D) and Joyce et al. PNAS 2014, report high beta-murocholic acid and Tauro-beta-murocholic acid in the liver bile acid pool of germ-free mice, when compared to colonized animals. While the liver levels of Tauro-beta-murocholic acid, Taurochenodeoxycholate and beta-murocholate seem to show a similar microbiome-dependent patterns in the day and night (Suppl. Fig. 7), we see elevation of Glycodeoxycholate in OligoMM12-colonized mice only in the dark cycle. In plasma, we see elevated beta-murocholate in OligoMM12-colonized mice as compared to germ-free mice during the light cycle but similar levels between germ-free and OligoMM12-colonized mice in the dark cycle, (Fig. 4E-F) potentially indicative of breakdown to beta-murocholic acid only during the phase when abundant food-derived carbon sources are available in the OligoMM12 microbiota. Therefore, the circadian rhythm-dependence of bile acid composition is highly specific to the tissue/fluid sampled, as well as to the microbiota composition. In order to not overlap with other groups investigating the functional consequences of bile acid hydrolases on the host, we have added a very brief description of these observations to the main text (Page 17, last paragraph).

5.      Calorie consumed in daytime vs. nighttime by GF mice appears to be different than those reported by others (PMID 25891358). Since the authors used a metabolic cage system it is likely that their results are more accurate. However, the authors should discuss these results in context of previous findings.

- Thank you for the reference. We confirmed that the caloric intake reported by Leone, et.al. is actually similar to the one reported by us. They reported approx. 15 to 16 Kcal/day for GF and 13Kcal for SPF in their control diet (Fig 1C) which are similar number to the ones we obtained (Fig 2F). We therefore do not see any discrepancy here.

6.     The authors state that "although [GF, Oligo] can equally fill up hepatic glycogen storages at the end of the dark phase, GF and OligoMM12 deplete hepatic glycogen faster during the light phase." However, don't these results contradict  their results in Fig 3A which shows that they are using non-glycogen (i.e., primarily fatty acid) sources of energy. How can the authors explain this discrepancy?

- Thank you for raising this interesting point. One of the major challenges of interpreting our results is that we cannot generate a complete map of the source of sugars/fats in the different mice over time, and clearly many things are changed between the germ-free and colonized animals with respect to energy absorption, storage and usage. It should be noted that the low RER shown in Fig. 3A shows less use of simple sugars, not specifically of liver glycogen, in germ-free mice. In SPF mice, it is an attractive hypothesis that the microbiome makes more simple sugars available to the host by continuously hydrolyzing complex carbohydrates. However, alternative hypotheses involving altered amino acid or short-chain fatty acid metabolism, or usage of glycogen stored in other tissues such as skeletal muscle could also potentially explain this observation. We should also point out that the RER in germ-free mice is never 0.7, indicating that some glucose metabolism is happening at all time-points examined, consistent with glycogen depletion. These caveats of this data interpretation have been added to the discussion (page 22, paragraph 1).

7.      Given how hard it is to bring a metabolic cage system into a germ-free environment, and this is a purported novelty in the paper, the authors should include in their supplementary figures quality control experiments that confirm that GF and OligoMM12 mice are indeed GF and OligoMM12 by the end of the experiment.

- Thank you for this excellent suggestion. Our new Suppl Fig 1A – Suppl Fig 1D now shows exactly these controls.

8.      Though the authors do a good job of placing their findings in context of previous studies, it's not immediately clear what the significance of Oligo treated mice being similar to GF mice in the dark but (slightly) similar to SPF mice during the day is (especially without knowing more about the dynamics of the Oligo microbiome).

- This is an important point. In many ways, what is most surprising is that OligoMM12-colonized mice, despite having 10^11^ bacteria per gram of cecal content, rather mimic the plasma and liver metabolome of germ-free mice than of fully colonized SPF animals. The slight reduction in distance between the SPF and gnotobiotic Liver metabolomes during the dark-phase is relatively unimpressive compared to this, indicating that although overall carbon-source usage seems to be normalized by the OligoMM12 microbiota during the dark-phase, there are many other aspects of the host-microbiota crosstalk that are not recapitulated by this simplified consortium. Having analyzed the composition of the OligoMM12 microbiome in the dark and light phase, there does not seem to be any major fluctuation in species composition over the circadian cycle, so the RER differences, as well as any difference in liver metabolome seem rather to relate to metabolic activities of the host and/or its colonizing species. The discussion of this data has been modified to now focus more on the similarity of OligoMM12 to germ-free mice (results page 15/16; discussion page 22)

MINOR COMMENTS

9.      TSE systems allow continuous measurement of EE. Is there a reason why hourly EE is not presented (as it is for RER and H2)? Area under the curve can be used for continuous time data. There may be additional insights gained by hourly EE.

- Thank you for this suggestion. We have now added raw EE vs time in showing daily variations over the course of the experiment (Supp Fig 3A). Overlaying this data show indeed that SPF mice have a higher raw EE during the dark-phase (Supp. Fig. 3B, and also Fig. 2A); however, this difference is counterbalanced by the higher lean mass observed in SPF mice; therefore, after normalization there are still no difference in EE among GF, OligoMM12 and SPF mice.

10.     Much of what is stated in the introduction, particularly in the last paragraph, should be shifted to the discussion. In particular, the authors should point out the novelty of having a metabolic cage system in the isolator which distinguishes their work from the others cited here (from my review no other one mentioned has done this).

- Thank you for this helpful suggestion. We have modified the text quite extensively in line with all of the reviewers’ suggestions and on top of this have moved (or removed) major sections from the introduction into the results and discussion sections, including the paragraph summarizing the paper’s findings.

11.     As with the previous comment, description of cecal mass contribution to body composition is important but should likely be in results or methods rather than the introduction.

- Thank you for this suggestion. The description of contribution of cecal mass to body composition have been moved to the results and discussion.

12.     The figures were extremely hard to see. However, this is likely due to the PDF-merge function of the journal since the supplementary figures were exceptionally clear. This has been noted to the editor to improve their software.

- Apologies for this difficulty. I hope it is resolved in the next system.

13.     The authors may want to use Canberra PCoA plots to look at distances between the metabolomics since this tool accounts for the relationship between metabolites (as opposed to plotting them as independent variables), which is increasingly being used instead of PCoA to determine differences between groups (<https://github.com/mwang87/q2_metabolomics>).

- We thank the review for this valuable comment. We switched from Euclidian PCA to Canberra PCoA for visualization and indeed the GF and Oligo group were better separated in Fig. 4A and B. We have adapted the text to describe the new visualization.

---------------------------------------

Reviewer #3: In this manuscript, the authors established a novel isolator-housed metabolic cage system to measure metabolic activities in germ-free and gnotobiotic mice with minimal contamination risk. Using this system, the authors observed no significant difference in energy extraction and expenditure among germ-free and SPF mice and mice with a defined microbiota (OligoMM12). The work goes on to demonstrate that mice with different microbiota groups have different circadian rhythms of respiratory exchange ratio, suggesting that the microbiota has an impact on the type of respiratory substrates in metabolism at different phases of a day/night cycle. The authors further showed differences in fat and glycogen accumulation, hydrogen and short-chain fatty acid production and host metabolome profiles in different microbiota groups.

Overall, the study is significant as it provides a new version of metabolic cage system for gnotobiotic research. It will be useful for understanding the roles of the microbiota in metabolic regulation. The authors identified multiple metabolic differences among the microbiota groups. How the metabolic reactions are connected and the molecular basis remain to be determined.

Specific comments:
1. Since the new system aims to minimize contamination for long-term examination, it would be important to provide results to show that germ-free mice remain sterile and OligoMM12 mice are not contaminated during and after experiment.

- Thank you for this excellent suggestion. Controls showing GF status of mice housed in the metabolic cages have been add in Suppl Fig 1A – Suppl Fig 1D. (text modification page 7)

2. In most cases, the authors showed averages of readouts by the metabolic cage system, which were very clear and informative. The authors have also included raw time-course readouts in the supplemental table. It would be very helpful if the authors could plot the time-course values to show the rhythmicity and consistency of measurements across multiple day-night cycles.

- Thank you for this suggestion. We have added in Suppl. Fig. 3A and B raw data of EE plotted over multiple days. Note that these data are not normalized to lean mass – this has been explicitly described in the associated text. When the higher lean mass of the SPF mice is used to normalize EE, any differences rapidly disappear. This data is now very briefly described in the results (page 9, paragraph 3).

3. The authors showed that OligoMM12 mice have more fat accumulation and different RER rhythmicity compared to germ-free and SPF mice. It would be very important if the authors could provide details of how they colonized the microbiota, such as how long the colonization was and what composition the microbiota was in their experiment. The gut microbiota is rhythmic and the rhythmicity may impact host rhythms. It would be helpful if the authors could examine the rhythm of OligoMM12 microbiota to gain some insight into the role of OligoMM12 microbiota in regulating host metabolic rhythms.

- Thank you for pointing out our lack of clarity here. All experiments were carried out with OligoMM12 mice that were colonized from birth, i.e., are part of an isolator-housed breeding colony stably colonized with this microbiota. This information has been added to the methods section “Animals” (page 24). In line with comments from other reviewers, we have now also carried out 16S amplicon analysis of the OligoMM12 microbiota in cecum content collected during the light phase and dark phase of the circadian cycle and could demonstrate no significant differences in cecal community composition or bacterial load between the two time-points. We therefore conclude that altered microbial metabolism over the circadian cycle is a result of phenotypic shifts in the activity of the microbes present rather than driven by compositional change.

4. The authors estimated energy contribution by fecal microbiota using pre-determined parameters including bacterial mass, bacterial density in feces and energy stored in bacteria. Did OligoMM12 mice and SPF mice have similar bacterial density in their feces so that the same parameters could be used for the estimation?

- This is an excellent comment. We have now measured bacterial density in the cecum content (which are normally in similar range to the observed bacterial densities in feces) on our OligoMM12 and SPF mice using bacterial flow (Supp. Fig. 4), as described in the methods. There is a significant difference in bacterial load (Oligo approx. 1.1x10^11 CFU/g vs SPF 1.6x10^11 CFU/g); which would translate into a difference in the contribution to energy density from 0.11 Kcal/g to 0.16Kcal/g. In the context of our calculations, this remains within the order of magnitude needed to explain the difference in energy density between germ-free and colonized mice and therefore does not change our overall conclusions. These updated numbers are now included in our calculations described in the results section (page 11, paragraph 2)

5. The authors showed that multiple amino acid metabolic pathways were enriched in the comparisons between OligoMM12 and GF and between OligoMM12 and SPF. Have authors seen differential abundances of corresponding amino acids besides Glycine, Serine, Threonine and Glutamate, as shown in Sup Fig 4?

- We have added a full list of all acquired amino acid graphs by targeted peak extraction (Suppl Fig. 7 and 8). It should be noted that the pathway enrichments consider both intermediates and final products of the pathways, and differences can be increased or reduced, such that altered flux through pathways is considered. Therefore, we urge caution with linearly translating between the boxplots showing single compounds and the pathway enrichments. Despite this, we do observe some consistent trends – for example “Glycine, Serine and Threonine metabolism” is enriched in germ-free versus OligoMM12-colonized mice comparisons in the light-phase and L-serine is also significantly decreased in the liver of OligoMM12-colonized mice at ZT5, but not ZT16. “Arginine biosynthesis” is enriched when comparing OligoMM12-colonized mice to germ-free, and L-arginine shows a trend to higher levels in liver of colonized mice than in germ-free. A sentence has been added to the text to describe these tendencies (page 16/17).
